# Organic solvent exposure and contrast sensitivity: comparing men and women

**DOI:** 10.1590/1414-431X20176568

**Published:** 2018-01-11

**Authors:** A.R. Oliveira, A.A. Campos, M.J.O. de Andrade, P.C.B. de Medeiros, N.A. dos Santos

**Affiliations:** 1 Departamento de Psicologia, Universidade Federal de Campina Grande, Campina Grande, PB, Brasil; 2 Departamento de Eletroeletrônica, Instituto Federal do Mato Grosso, Cuiabá, MT, Brasil; 3 Departamento de Psicologia, Universidade Federal da Paraíba, João Pessoa, PB, Brasil; 4 Departamento de Psicologia, Universidade Federal do Piauí, Parnaíba, PI, Brasil

**Keywords:** Visual perception, Spatial frequencies, Neurotoxic agents, Chronic exposure, Gender

## Abstract

The goal of this study was to compare the visual contrast sensitivity (CS) of men and women exposed and not exposed to organic solvents. Forty-six volunteers of both genders aged between 18 and 41 years (mean±SD=27.72±6.28) participated. Gas station attendants were exposed to gas containing 46.30 ppm of solvents at a temperature of 304±274.39 K, humidity of 62.25±7.59% and ventilation of 0.69±0.46 m/s (a passive gas chromatography-based sampling method was used considering the microclimate variables). Visual CS was measured via the psychophysical method of two-alternative forced choice using vertical sinusoidal gratings with spatial frequencies of 0.2, 0.5, 1.0, 2.0, 5.0, 10.0, and 16.0 cpd (cycles per degree) and an average luminance of 34.4 cd/m^2^. The results showed that visual CS was significantly lower (P<0.05) in the following groups: i) exposed men compared to unexposed men at frequencies of 0.2, 0.5, 1.0, and 2.0 cpd; ii) exposed women compared to unexposed women at a frequency of 5.0 cpd; and iii) exposed women compared to exposed men at a frequency of 0.5 cpd, even at exposures below the tolerance limit (300 ppm). These results suggest that the visual CS of exposed men was impaired over a wider range of spatial frequencies than that of exposed women. This difference may have been due to the higher body fat content of women compared to that of men, suggesting that body fat in women can serve as a protective factor against neurotoxic effects.

## Introduction

Organic solvents are typically present in paints, adhesives, glues, plastics and fuels (1). Automotive gasoline, for example, is made up of a complex mix of aromatic hydrocarbons, or BTEX (benzene, toluene, ethylbenzene, and xylene), the major properties of which include volatilization and lipophilicity (2). BTEX is toxic, and its effects have been demonstrated in experimental, clinical and epidemiological studies (3,4). Research findings indicate that chronic occupational exposure to these compounds alters the function of multiple organs and systems, including the central nervous system (CNS); this alteration can lead to losses in basic visual functions, including the contrast sensitivity function (CSF) (5). The CSF is a classical measure used to assess the response of the human visual system to a wide range of spatial frequencies and to measure an individual's ability to describe the visual perception of a given pattern (object) at different levels of contrast or brightness (6). The CSF is a non-invasive and objective measure that has been used due to its effectiveness in detecting changes that may occur even prior to the emergence of evident clinical phenomena (7).

Although a number of studies demonstrating that chronic exposure to organic solvents affects visual processing and basic visual functions such as CSF have been conducted, the majority of the studies on this topic have been performed in men (8,9) rather than in women, despite the evidence that men and women have different body compositions, a factor that may influence the toxicokinetics of solvents (10). Thus, the results of these studies have been generalized to women (11), for whom the same tolerance limits are then recommended based on studies conducted with men alone (12). This bias may have occurred due to the difficulty in finding appropriate samples of women who have been exposed to organic solvents, given that most workers exposed to such conditions are male (13).

Due to the toxicokinetics and toxicodynamics of the toxic agents involved, exposure to organic solvents can trigger different behavioral and neurophysiological responses depending on gender (14). This occurs because men and women differ in some compositional and physiological parameters, such as the levels of enzymes and specific hormones, water volume, lean mass and body fat (10). For example, women generally have more fat mass than men, and men have more lean mass than women (15,16). In this context, it is important to investigate the gender-related effects of organic solvents on CSF (17) because this issue has not been addressed (18) except in a single study by Böckelmann et al. (19).

Böckelmann et al. (19) measured the CSF using the Vision Contrast Test System VCTS 6500 with spatial frequencies of 1.5, 3.0, 6.0, 12.0, and 18.0 cpd of visual angle and a luminance of 100 cd/m^2^. The maximum workplace concentration index (*I*_mak_) in that study ranged between 0.02 and 0.76, values that are below the tolerance value of 1.0. Nonetheless, the exposed men had lower values of visual CSF than the unexposed men at spatial frequencies of 3.0 and 18.0 cpd in the right eye and at spatial frequencies of 1.5, 3.0, and 6.0 cpd in the left eye (19). The group of exposed women displayed smaller visual CSF values than the group of unexposed women at spatial frequencies of 3.0 cpd in the right eye and at spatial frequencies of 1.5, 6.0, and 12.0 cpd in the left eye (19). Moreover, the men and women in the study showed different visual CSF values, but there was no correlation between gender and exposure (15).

It is important to understand gender-based differences in the sensory responses of workers pre-exposed to neurotoxic substances as a basis for developing risk assessment strategies that maintain and protect the health of both men and women, especially considering that women may be pregnant or breastfeeding during exposure to solvents (15). Thus, considering that the visual CSF has been shown to be a good indicator of neurotoxic changes arising from the effects of organic solvents and that men and women may respond differently to organic solvents as a result of their physical and physiological constitutions, this study aimed to measure and compare the visual CSF of 1) men with and without history of exposure to organic solvents; 2) women with and without history of exposure to organic solvents, and 3) women and men who were exposed to organic solvents (based on the level of exposure to solvent vapors and microclimate conditions). This study started from the hypothesis that organic solvents differentially affect the visual CSF of exposed men versus that of exposed women.

## Material and Methods

### Participants

A total of 24 gas station attendants and 22 control subjects were recruited by convenience. The study group was formed by participants working at gas stations located in the four zones (South, North, East, West) of the city of João Pessoa, Paraíba, Brazil. All participants read and accepted the written informed consent after the first interview. The control groups were composed of university workers, with similar age groups and education levels.

The volunteers were divided into the following groups: male study group (SGm), male control group (CGm), female study group (SGf), female control group (CGf).

The gas station workers worked 6 days per week and 8 h a day, with a 1-h lunch break. The gas stations sell ethanol, gasoline and diesel.

Initially, 54 participants were included in the study but 8 were later excluded; these included 2 SGf and 2 SGm individuals who reported engaging in regular physical activity, and 2 CGm individuals (one who reported amblyopia, that is, a change in spatial vision resulting from abnormal binocular interaction during the critical period of development (20), and another who had previously worked in a gas station).

Study inclusion and exclusion criteria were as follows: 1) Inclusion criteria, study groups: at least 6 months of exposure to organic solvents; normal or corrected visual acuity of 20/20; and having work-related activities during the morning or afternoon shifts (8,21). 2) Inclusion criteria, control groups: no history of exposure to chemicals and normal or corrected visual acuity. 3) Exclusion criteria, study groups (SG): individuals who reported exposure to chemical vapors prior to working at the gas station (22) and those who regularly used personal protective equipment (PPE); both groups: chronic smoking, alcohol abuse and other drugs, prior diagnosis of eye, neurological or psychiatric disease, diabetes, cardiovascular disease such as hypertension, and regular physical activity, i.e., a minimum of 30 min of physical activity three or more times per week (23).

### Instruments

#### Achromatic assessment

The stimuli used to test visual acuity was generated by the Metropsis software (Cambridge Research Systems, UK) using a 19-inch monitor with 1,024×786 pixels and a sampling rate of 100 Hz. A Visual Stimulus Generator (*ViSaGe*) system with a VSG 2/5 video card (Cambridge Research Systems), and a Dell Precision T3500 computer (Cambridge Research Systems) with a W3530 processor were used. The average luminance used was 34.4 cd/m^2^, and vertical sinusoidal gratings at spatial frequencies of 0.2, 0.5, 1.0, 2.0, 5.0, 10.0, and 16.0 cpd were used as visual stimuli. These frequencies were selected based on other study results (24,25).

#### Sociodemographic questionnaire

The participants were questioned regarding their age, education level, consumption of alcohol, tobacco and other drugs, the presence of eye or neurological diseases, diabetes and hypertension, and, specifically for the study groups, length of service, work hours per day and week (in hours), and use of PPE.

#### Rasquin's chart of "E" optotypes for assessing visual acuity

These charts provide a directional test that consists of an optotype ("E") that varies in position (facing up, down, left or right) and size from row to row. The role of the participant was to identify the open side of the "E". An acuity of 6/6 or 20/20 (equivalent to 20 feet) is considered normal, indicating that an observer is able to identify an object at a distance of 6 m.

### Passive sampling

A vapor monitoring badge or passive sampler (Organic Vapor Monitor-OVM 3500, 3M, Brazil) containing activated charcoal was used to estimate the daily average concentration of gasoline vapors at different gas stations. This method is cheap and shows efficiency to identify vapor concentrations. The data collection occurred over a 20-day period for 7 continuous hours per day. The material was stored in appropriate bottles composed of OVM 3500. The alcohol concentration measurement was not included because alcohol contain fuels used less frequently in Brazil.

The measuring of gasoline vapor concentration involved the following steps: 1) breaking the seal of the metal dosimeter storage box; 2) recording information on the back of the dosimeter; 3) attaching the dosimeter near the gas station attendant's breathing height; 4) monitoring the participant during his or her entire shift; 5) removing the white filter and tightly sealing it with the provided plastic lid, and 6) storing the filter in the metal box and sending it to the laboratory for hydrocarbon gas chromatography. This analysis is recommended in the standard 1550 of the National Institute for Occupational Safety and Health and was performed by SGS - Environ, a world leader in inspection, verifications, testing and certification (http://www.sgsgroup.com.br/pt-BR/Health-Safety/Quality-Health-Safety-and-Environment/Health-and-Safety/Occupational-and-Industrial-Hygiene.aspx).

### Microclimate assessment

The temperature, humidity and ventilation of the exposure area were measured to evaluate the influence of the environment surrounding the stations on the emission of organic vapors. The temperature and relative humidity of the air were measured using an HT-200 digital thermo-hygrometer with a precision of ±274.15 K and a measurement scale of -293.15 to 343.15 K (Instrutherm, Brazil). The wind speed was measured using an AD-250 digital anemometer, (Instrutherm) with a scale of 0.4 to 30 m/s and an accuracy of 0.1 m/s. The measurements were made at the beginning, middle and end of the work day, and then the day average was calculated.

### Procedure

The study group participants were recruited from the gas stations where they worked after authorization from the Union of Oil Products Retailers, Paraíba State, Brazil. The control participants were recruited from the general population. The groups were matched by gender, age and education level to eliminate or reduce the effects of these variables on the results.

A cross-sectional, quasi-experimental, *ex post facto* design was used. The visual acuity of all participants was assessed before the visual contrast sensitivity (CS; 1/threshold) was measured. The contrast sensitivity was log-transformed (log 10). The contrast threshold was measured using the psychophysical method of two-alternative forced choice in which the spatial frequency (vertical sinusoidal grating) was varied; the method was adapted from Wetherill and Levitt (26). The procedure for measuring the threshold for detection of each frequency consisted of simultaneously presenting a pair of stimuli on the screen and asking the participant to choose which of the two stimuli represented that frequency by pressing the left or right button of the answer key (CT6 model, Cambridge Research Systems). The stimuli were squares with five degrees of visual angle and were presented on the screen at a position 2.5 degrees between the center of the screen and the edge.

This procedure uses the descending and ascending variation found in the staircase method as the criterion for measuring the visual threshold. This method is based on calculating the probability of the participant making consecutive hits, that is, in approximately 100 presentations with two stimuli to choose from (test stimulus and neutral stimulus), the spatial frequency (test stimulus) is perceived by the participant approximately 79% of the time.

The test began by presenting contrast values at a suprathreshold level to facilitate the task execution. Next, staircase reversal criteria were adopted in which three hits were required to reduce the contrast and one error was required to increase it. Thus, after three consecutive hits, the contrast decreased by 0.7 decibels (dB), and, with every wrong answer, the contrast increased by 1.0 dB. After eight reversals (maximum and minimum thresholds) were recorded for each spatial frequency, the experiment was concluded. Visual CS measurements were obtained with the participant positioned 150 cm from the monitor screen with binocular vision and natural pupils. The binocular measurement of visual perception is justified based on its use in other studies using samples of gas station attendants (24,25). Another reason for binocular measurement is that there are no differences between binocular and monocular measurements. For example, the study by Costa et al. (27) showed that the results obtained in the binocular and monocular conditions did not differ statistically for the thresholds measured in the Trivector and Ellipse procedures of the Cambridge Color Test.

The sinusoidal spatial frequencies 0.2, 0.5, 1.0, 2.0, 5.0, 10.0, and 16.0 cpd of visual angle and an average luminance of 34.4 cd/m^2^ were used. Each stimulus was presented for 2 s with a one-second interval between each stimulus and a 3-s interval between attempts. The experimental sessions varied according to errors and hits of each participant; the average session duration was 25 min.

The present study followed the ethical principles of the Declaration of Helsinki and was approved by the local Ethics Committee (Centro de Ciências da Saúde, Universidade Federal da Paraíba, No. 21350113.9.0000.5188). The participants signed an informed consent form. This research met the requirements of Resolution 466/12 of the National Health Council (28), which establishes guidelines and regulatory standards for human research in line with international guidelines.

### Statistical analysis

Descriptive and inferential statistical analyses were carried out with SPSS (Statistical Package for the Social Sciences, USA), version 20. Data were checked for normality (Kolmogorov-Smirnov and Shapiro-Wilk) and homogeneity of variance (Levene), indicating the use of parametric analyses for comparison of age, education level (one-way ANOVA) and exposure time (*t*-test), and of non-parametric analyses for comparison of CS (Mann-Whitney) between the groups.

## Results

The sociodemographic data are presented in Table 1. No significant differences were observed in terms of age (F(3,42)=1.08, p=0.37) or education level (F(3,42)=0.55, P=0.65) between the four groups, or for exposure time between exposed women and exposed men (t(22)=1.02, P=0.32).

**Table 1.** Sociodemographic data of study and control male groups (SGm and CGm) and study and control female groups (SGf and CGf).

Sociodemographic data	SGm (n=12)	CGm (n=11)	SGf (n=12)	CGf (n=11)
Age (years)	30±6.48	26.7±6.50	26.9±5.86	26.5±6.50
Education (years)	9.83±2.04	10.33±0.89	9.73±1.85	10.45±1.51
Number of working years	6.53±4.38	-	4.70±3.53	-

Data are reported as means±SD.

A repeated-measures design was used to estimate the sensory thresholds of the participants. This type of study usually involves a small number of participants and uses several measures of the same participants obtained at different times (29,30).

### Gasoline concentration and microclimate assessment

The daily average concentrations of organic vapors were obtained using the OVM. Table 2 presents the average values of the gasoline concentration and microclimate variables over 20 days of sampling at the gas stations. The analysis procedure to obtain a value for gasoline was carried out through laboratory tests performed by the SGS group. According to company regulations for gasoline, turpentine and kerosene, the NIOSH 1550 method is used (Environ IT.10-424 - Selected Organic Vapors - Determination in Atmospheric Air) - Gas Chromatography with Flame Ionization Detector (for more information visit: http://www.sgsgroup.com.br/pt-BR/Health-Safety/Quality-Health-Safety-and-Environment/Health-and-Safety/Occupational-and-Industrial-Hygiene.aspx).

**Table 2.** Passive exposure to solvents and microclimatic evaluations during 7 h of activity in the 20-day period.

Parameter	Median	Maximum	Minimum
Passive exposure			
Gasoline (ppm)	8.35	46.30	4.60
Microclimatic evaluation			
Temperature (°C)	31.00	306.00	301.00
Humidity (%)	60.50	79.00	52.00
Ventilation (m/s)	0.60	1.90	0.22

ppm: parts per million; m/s: meters per second.

The results showed that the maximum daily exposure level (46.30 ppm) was below the threshold value adopted by the American Conference of Governmental Industrial Hygienists - ACGIH of 300 ppm for climate stability over the 20 days of measurements. Little variation in temperature, humidity and ventilation was observed.

### Contrast sensitivity

The mean visual CS values are reported in Table 3.

**Table 3.** Visual contrast sensitivity function scores by spatial frequency in cpd and by group (exposed and unexposed women or exposed and unexposed men).

Frequency (cpd)	SGm (n=12)	CGm (n=11)	P	SGf (n=12)	CGf (n=11)	P	SGm x SGf
0.2	164.43±160.79	174.50±153.22	0.04*	108.56±65.46	111.126±37.76	0.07	0.90
0.5	116.43±38.20	197.20±73.60	0.01**	167.12±67.47	225.45±79.77	0.07	0.04*
1.0	202.68±97.20	252.94±60.27	0.04*	245.48±147.74	220.37±70.47	0.71	0.41
2.0	355.36±114.80	447.15±133.66	0.04*	413.98±184.90	350.02±153.21	0.26	0.35
5.0	285.98±115.52	509.00±493.48	0.05	275.34±172.40	452.60±176.18	0.04*	0.68
10.0	109.19±45.95	212.66±173.14	0.08	185.32±158.00	207.04±119.97	0.44	0.22
16.0	34.23±12.95	45.45±20.84	0.17	43.70±42.69	36.99±19.66	0.58	0.45

Data are reported as means±SD. SGm: male study group; CGm: male control group; SGf: female study group; CGf: female control group.

* P<0.05;

** P<0.01 (Mann-Whitney test).

**Figure 1.**
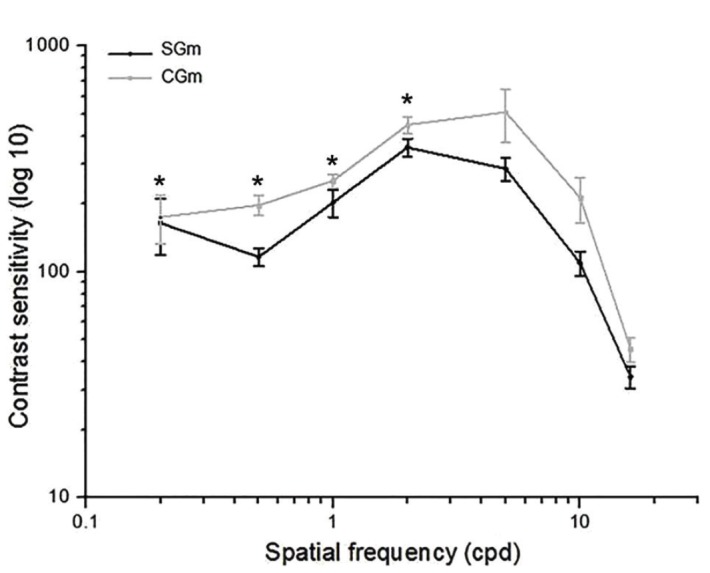
Male study group (SGm) and male control group (CGm) contrast sensitivity curve. The data are reported as means and the vertical lines indicate the standard error of the mean for each spatial frequency (0.2, 0.5, 1.0, 2.0, 5.0, 10.0 and 16.0 cpd). *P<0.05; **P<0.01 (Mann-Whitney test).

The SGm group presented lower visual CSF than the CGm group at all frequencies tested, with significant differences at the following frequencies: 0.2 cpd; 0.5 cpd; 1.0 cpd; and 2.0 cpd. The SGm group was 1.7, 1.1, 1.3 and 1.2 times more sensitive than the CGm group at the frequencies of 0.5, 0.2, 2.0 and 1.0 cpd, respectively. These results are included in Figure 1, which shows the mean SGm and CGm contrast sensitivity values as a function of the spatial frequency.

These results suggest impaired visual CSF of varying degrees in SGm compared to CGm for the frequency band 0.2-2.0 cpd. The observed differences between the two groups appeared to be robust because the exposed and unexposed men were matched by age and education level.

**Figure 2.**
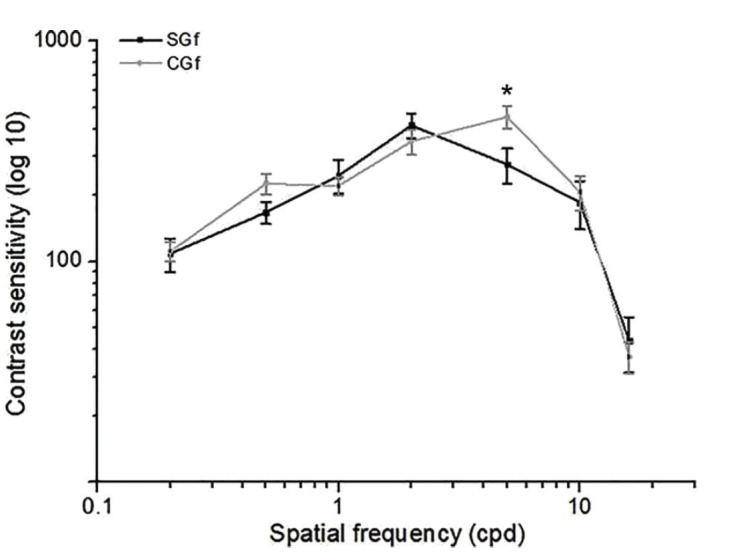
Female study group (SGf) and female control group (CGf) contrast sensitivity curve. Data are reported as the means and vertical lines show the standard error of the mean for each spatial frequency (0.2, 0.5, 1.0, 2.0, 5.0, 10.0 and 16.0 cpd). *P<0.05 (Mann-Whitney test).

The SGf group presented lower visual CSF than the CGf group at four frequencies, with a significant difference for 5.0 cpd as shown in Figure 2, in which the mean SGf and CGf contrast sensitivity values are plotted as a function of spatial frequency. The SGf group was 1.6-fold less sensitive than the CGf group at this frequency.

Considering that these groups were homogeneous in terms of age and education level, this result indicated that exposure to organic solvents affected visual CSF at the frequency of 5.0 cpd in exposed women.

**Figure 3.**
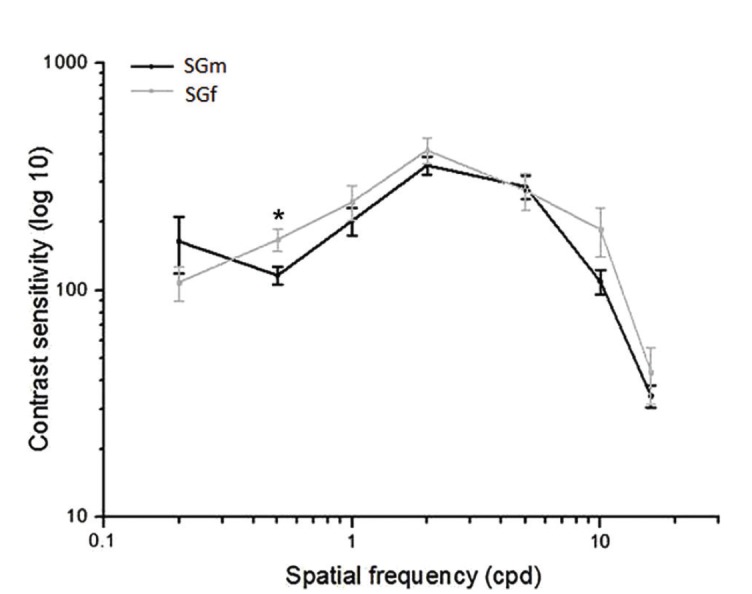
Male study group (SGm) and female study group (SGf) contrast sensitivity curve. Data are reported as means and vertical lines show the standard error of the mean for each spatial frequency (0.2, 0.5, 1.0, 2.0, 5.0, 10.0, and 16.0 cpd). *P<0.05 (Mann-Whitney test).

The results demonstrated that exposed women and men showed different patterns of changes in CSF. Figure 3 shows the mean SGf and SGm contrast sensitivity values as a function of spatial frequency.

In general, women were 1.7, 1.4, 1.3, 1.2 and 1.2 times more sensitive than men at the frequencies of 10.0, 0.5, 16.0, 1.0 and 2.0 cpd, respectively. However, at the frequencies of 0.2 and 5.0 cpd, men were approximately 1.5 and 1.0 times more sensitive, respectively, than women.

Finally, correlation analysis between the average concentration of gasoline vapor measured and the CS of the SGf and SGm groups were conducted. Spearman's correlation did not show significant results.

## Discussion

The primary goal of this study was to measure visual CS in men and women who had or had not undergone chronic occupational exposure to organic solvents while accounting for the level of exposure to gasoline vapors and defined microclimate conditions. Previous studies have not investigated whether gender is related to differences in the effects of organic solvents on visual CS.

### Visual CS of exposed men and control men

Exposed men showed lower CS than control men at low and medium cpd frequencies. These results corroborate those of most other studies (8,9,19,24,31–33). For example, Costa et al. (24) found differences for the frequencies of 0.2, 1.0, 2.0, 5.0, 10.0, and 20.0 cpd of visual angle but not for 0.5 cpd. Mergler (13) found changes in CS for spatial frequencies of 1.2, 3.0, 6.0, and 12.0 cpd but not for 18 cpd. However, the results of these studies differ from the results of the study by Lacerda et al. (25) regarding the frequency bands affected by pre-exposure to organic solvents; in that study, differences in visual CS at the high frequencies of 20 and 30 cpd were found.

Some basic differences preclude a direct comparison of our study to that by Lacerda et al. (25). In that study, the authors used the "Yes-No" psychophysical adjustment method, 43.5 cd/m^2^ average luminance, exposure times ranging from 1 month to 22 years and did not perform microclimate assessment of the vapor level in the work environment. This may partly explain why they found differences in visual CS at spatial frequency bands slightly different from those in which differences were found in our study.

The results of the present study show that although the level of exposure was below the threshold adopted by the ACGIH (300 ppm) and the climate conditions were stable (Table 2), exposed men presented lower CS than that of controls. This may be a reflection of neural changes caused by exposure to organic solvents.

### Visual CS of exposed women and control women

Exposed women presented lower CS than that of control women at the frequency of 5.0 cpd. This result demonstrates that changes occur in the CSF of women exposed to organic solvents, corroborating the results of a study by Böckelmann et al. (19), which showed that women were less sensitive at spatial frequencies of 1.5, 6.0, and 12 cpd in the left eye and at the frequency of 3.0 cpd in the right eye. Those results were obtained even at the maximum concentration indices of the substances (I_MAK_ limit 0.02 to 0.76) allowable within the tolerance limits (1.0) of the country (Germany). It is important to note that a direct comparison between the present study and the study by Böckelmann et al. (19) cannot be made because different psychophysical methods and luminance conditions to measure CSF were used. The participants in that study had greater exposure time and consumed alcohol and tobacco. Moreover, physical activity and microclimate conditions of that study were not considered. Nonetheless, the results of both studies showed that changes occur in the CSF of women exposed to organic solvents.

No other study comparing the CS of women exposed to organic solvents was found. However, a study with the children of women who were exposed to solvents during pregnancy showed a significant reduction in CS at low and intermediate spatial frequency bands compared with children in the control group (34). These results are important because they suggest that prenatal exposure to solvents is associated with deficits in CS. Therefore, the current occupational exposure limits for pregnant women must be reassessed (34).

### Visual CS of exposed women and exposed men

According to visual CS, exposed men were less sensitive than the exposed women, specifically at the frequency of 0.5 cpd. These results corroborate the results obtained by Mergler et al. (35–37), which suggest that there are gender-related differences in the visual responses of individuals exposed to organic solvents. This difference was expected because some authors have stated that toxicokinetic and toxicodynamic processes can lead to specific neurotoxic responses that are related to the biological differences between men and women (15,16).

These results are important because they revealed significant differences between exposed men and women at a stimulus frequency of 0.5 cpd. Our findings differ from the results of the study by Böckelmann et al. (19), in which there was no correlation between gender and exposure (exposed and controls). These discrepant results might be explained by differences in the sample characteristics, visual stimuli and luminance used in the two studies.

Overall, the results of the present study indicated that in women CSF was less affected by exposure to organic solvents than in men. This finding raises the possibility of women having a factor that protects against the neurotoxic effects of organic solvents, such as increased body fat content (16,17). Some toxic agents selectively accumulate in specific parts of the body as a result of their high fat solubility (38,39). Thus, when organic solvents enter the blood circulation, their plasma concentration decreases as they pass through regions with greater fat density, hindering their absorption by the CNS. Thus, CS in women might be less affected or affected in a different manner than in men depending on the type of solvents and the exposure time.

In conclusion, the results obtained in the present study suggest that occupational exposure to organic solvents has differential effects on the basic visual functions (CS) of men and women. The results indicate that women who are exposed to organic solvents are less affected than men. These changes are probably related to the physiological characteristics of men and women, which may cause their bodies to interact differently with organic solvents, producing different toxicokinetics.

Considering that the findings regarding organic solvent effects in the visual system are part of preliminary studies, the results must be interpreted with caution. Even with the adopted methodological rigor, some limitations were present such as the lack of skin evaluations and the density of the retinal macula, the possibility of undiagnosed ophthalmological alterations prior to the study, as well as the sample size, which are factors that may be related to the complexity of the results. Also, it is important to point out that because no comparison was carried out between obese and lean subjects of the same gender, we cannot attribute the observed difference to gender.

From this perspective, it is important that further studies investigate aspects such as the retina, the lens opacities, presence of any pathology in the anterior segment of the eye or in any part of the eye that could affect the results. In addition, studies should evaluate the risk of skin penetration, as well as the role of body composition (e.g., fat and lean tissue) on the effects of organic solvents on basic visual perception and on other sensory functions as well as the neurocognitive and biological effects that exposure to these solvents has on women and men. Moreover, it is important to investigate whether the performance of women varies according to the ovulation cycle phases because recent studies suggest that cognitive and emotional processing of visual stimuli in women may be more or less similar to that of men depending on the cycle phase (40). Thus, additional studies in which these variables are addressed may help clarify the results of neurotoxicology studies that compare men and women visual CS results.
